# A checklist for clinical trials in rare disease: obstacles and anticipatory actions—lessons learned from the FOR-DMD trial

**DOI:** 10.1186/s13063-018-2645-0

**Published:** 2018-05-10

**Authors:** Rebecca A. Crow, Kimberly A. Hart, Michael P. McDermott, Rabi Tawil, William B. Martens, Barbara E. Herr, Elaine McColl, Jennifer Wilkinson, Janbernd Kirschner, Wendy M. King, Michele Eagle, Mary W. Brown, Deborah Hirtz, Hanns Lochmuller, Volker Straub, Emma Ciafaloni, Perry B. Shieh, Stefan Spinty, Anne-Marie Childs, Adnan Y. Manzur, Lucia Morandi, Russell J. Butterfield, Iain Horrocks, Helen Roper, Kevin M. Flanigan, Nancy L. Kuntz, Jean K. Mah, Leslie Morrison, Basil T. Darras, Maja von der Hagen, Ulrike Schara, Ekkehard Wilichowski, Tiziana Mongini, Craig M. McDonald, Giuseppe Vita, Richard J. Barohn, Richard S. Finkel, Matthew Wicklund, Hugh J. McMillan, Imelda Hughes, Elena Pegoraro, W. Bryan Burnette, James F. Howard, Mathula Thangarajh, Craig Campbell, Robert C. Griggs, Kate Bushby, Michela Guglieri

**Affiliations:** 1 John Walton Muscular Dystrophy Research Centre, Institute of Genetic Medicine, Newcastle University, Newcastle upon Tyne, NE1 3BZ UK; 2 University of Rochester Medical Center, Rochester, NY USA; 3 Newcastle University, Newcastle upon Tyne, UK; 4 University Medical Center, Freiburg, Germany; 5 National Institutes of Health, Bethesda, MD USA; 6 UCLA, Los Angeles, CA USA; 7 Alder Hey Children’s Hospital, Liverpool, UK; 8 Leeds Teaching Hospitals, Leeds, UK; 9 GOSH, UCL, London, UK; 10 Neurological Institute “Carlo Besta”, Milan, Italy; 11 University of Utah, Salt Lake City, UT USA; 12 Greater Glasgow and Clyde NHS Yorkhill Hospital, Glasgow, UK; 13 Birmingham Heartlands Hospital, Birmingham, UK; 14 Nationwide Children’s Hospital, Columbus, OH USA; 15 Ann and Robert H. Lurie Children’s Hospital, Chicago, IL USA; 16 University of Calgary, Calgary, Canada; 17 University of New Mexico, Albuquerque, NM USA; 18 Boston Children’s Hospital, Boston, MA USA; 19 Children’s Hospital, Technical University Dresden, Dresden, Germany; 20 University of Essen, Essen, Germany; 21 Children’s University Hospital, Göttingen, Göttingen, Germany; 22 University of Torino, Turin, Italy; 23 UC Davis Medical Center, Sacramento, CA USA; 24 University of Messina AOU Policlinico Gaetano Martino, Messina, Italy; 25 University of Kansas Medical Center, Kansas City, KS USA; 26 Nemours Children’s Hospital, Orlando, FL USA; 27 Penn State College of Medicine, Hershey, PA USA; 28 Children’s Hospital of Eastern Ontario, Ottawa, Canada; 29 Royal Manchester Children’s Hospital, Manchester, UK; 30 University of Padova, Padua, Italy; 31 Vanderbilt Children’s Hospital, Nashville, TN USA; 32 University of North Carolina School of Medicine, Chapel Hill, NC USA; 33 Children’s National Medical Center, Washington, DC USA; 34 Children’s Hospital London Health Sciences Centre, London, Canada

**Keywords:** Clinical trial, Academic-led clinical trial, Clinical trial regulations, Duchenne muscular dystrophy, Rare disease

## Abstract

**Background:**

Trials in rare diseases have many challenges, among which are the need to set up multiple sites in different countries to achieve recruitment targets and the divergent landscape of clinical trial regulations in those countries. Over the past years, there have been initiatives to facilitate the process of international study set-up, but the fruits of these deliberations require time to be operationally in place. FOR-DMD (Finding the Optimum Steroid Regimen for Duchenne Muscular Dystrophy) is an academic-led clinical trial which aims to find the optimum steroid regimen for Duchenne muscular dystrophy, funded by the National Institutes of Health (NIH) for 5 years (July 2010 to June 2015), anticipating that all sites (40 across the USA, Canada, the UK, Germany and Italy) would be open to recruitment from July 2011. However, study start-up was significantly delayed and recruitment did not start until January 2013.

**Method:**

The FOR-DMD study is used as an example to identify systematic problems in the set-up of international, multi-centre clinical trials. The full timeline of the FOR-DMD study, from funding approval to site activation, was collated and reviewed. Systematic issues were identified and grouped into (1) study set-up, e.g. drug procurement; (2) country set-up, e.g. competent authority applications; and (3) site set-up, e.g. contracts, to identify the main causes of delay and suggest areas where anticipatory action could overcome these obstacles in future studies.

**Results:**

Time from the first contact to site activation across countries ranged from 6 to 24 months. Reasons of delay were universal (sponsor agreement, drug procurement, budgetary constraints), country specific (complexity and diversity of regulatory processes, indemnity requirements) and site specific (contracting and approvals). The main identified obstacles included (1) issues related to drug supply, (2) NIH requirements regarding contracting with non-US sites, (3) differing regulatory requirements in the five participating countries, (4) lack of national harmonisation with contracting and the requirement to negotiate terms and contract individually with each site and (5) diversity of languages needed for study materials. Additionally, as with many academic-led studies, the FOR-DMD study did not have access to the infrastructure and expertise that a contracted research organisation could provide, organisations often employed in pharmaceutical-sponsored studies. This delay impacted recruitment, challenged the clinical relevance of the study outcomes and potentially delayed the delivery of the best treatment to patients.

**Conclusion:**

Based on the FOR-DMD experience, and as an interim solution, we have devised a checklist of steps to not only anticipate and minimise delays in academic international trial initiation but also identify obstacles that will require a concerted effort on the part of many stakeholders to mitigate.

**Electronic supplementary material:**

The online version of this article (10.1186/s13063-018-2645-0) contains supplementary material, which is available to authorized users.

## Background

The research and patient communities are united in the opinion that rare diseases (defined in the USA as diseases affecting fewer than 200,000 people at any given time and in the European Union (EU) as diseases affecting fewer than 5 people in 10,000 [1]) require new and better therapies [2]. A tangible sign of the commitment to this cause is that an international consortium of funders has been set up to ensure that financial resources will be in place to develop and deliver 200 new therapies for rare diseases by 2020 [2]. Drug development programmes in rare diseases have many challenges [3], some of which differ from those facing researchers working on common diseases: the lack of clinical and research-savvy experts, which results in difficulties in setting up and running studies at inexperienced sites and the scarcity of patients, which means that large-scale studies will always require the set-up of multiple centres in different countries to achieve recruitment targets for proof-of-efficacy clinical trials.

Academic-led studies have the additional challenge of budgetary and capacity constraints [4]. As a result, it is difficult for academic-led studies to access contracted research organisations (CROs). These organisations have an international infrastructure of regulatory specialists and are delegated the responsibility of gaining regulatory and site approvals, avoiding inexperienced investigators and their study teams having to learn and navigate the processes themselves, therefore aiding multi-centre trial set-up [5]. In both rare and common diseases, clinical trials with multiple sites in different countries pose many challenges due to the diversity, both between and within nations, with reference to care standards [5], laws, regulations and guidelines governing clinical trials [5–7]. The incompatibility between countries’ policies when setting up international studies and the consequent bureaucratic delays has been highlighted with specific reference to North America and Europe [7] and National Institutes of Health (NIH)-funded studies [5, 6, 8].

However, international, multi-centre studies provide an added value by promoting global standards of care and expansion of the market for new treatments; this is particularly apparent in rare diseases due to the limited number of experts [4, 9, 10]. In addition, academic-led studies hold their own value [7, 11], often focussing on ideas to improve patient care that may not have as much financial gain to the pharmaceutical industry, like drug repurposing or the method in which a drug is administered, as in the case of FOR-DMD (Finding the Optimum Steroid Regimen for Duchenne Muscular Dystrophy).

In recognition of the importance and challenge of international studies, there have recently been initiatives to standardise and harmonise the regulatory aspects of clinical research worldwide. This has been occurring in both our area of expertise: Duchenne muscular dystrophy, a rare neuromuscular disease affecting mainly boys, and in more general regulatory fields. The EU has awarded funding to disease-specific networks, such as TREAT-NMD, a neuromuscular network to provide the infrastructures to ensure efficient delivery of new therapies to patients [12], and generic networks, such as ECRIN (European Clinical Research Infrastructure Network) [13] and EATRIS (European Infrastructure for Translational Medicine) [14], in order to support trial development. Moreover, in 2004, the Voluntary Harmonisation Procedure [15] was developed in the EU to streamline the review process for competent authority applications for multi-centre clinical trials, recognising this challenge. However, as the name suggests, there is no legal obligation for EU countries to take part in this process, which has had limited application so far [16]. The release of the updated EU clinical trial regulations [17], due for implementation in 2017, aims to further streamline the regulatory system in all EU countries in a binding legislative act, to ease the burden for multi-centre, international studies [18].

In the USA, efforts to reduce bureaucratic delays have been introduced with NeuroNEXT (Network for Excellence in Neuroscience Clinical Trials), an organisation set up by the National Institute of Neurological Disorders and Stroke to increase the efficiency of clinical trials and expand the capability to promptly deliver new therapies for neurological disorders to patients [19]. NeuroNEXT has created a centralised system, which ensures a single human subject/ethics review, contracting process, coordination centre, data management and statistics centre for multi-centre studies [20]. In addition, the proposed changes to the Common Rule (Federal Policy for the Protection of Human Subjects) [21] set out to streamline approval processes for multi-centre clinical trials in order to avoid the widely recognised, unnecessary regulatory reviews and lack of efficiency in the current system [22, 23]. However, both the NeuroNEXT initiative and the proposed changes to the Common Rule focus on US multi-centre trials and do not apply outside the USA.

Harmonisation between the USA and EU countries in terms of regulatory requirements and law has not been achieved yet, and, therefore, setting up clinical trials across the EU and USA remains challenging [6, 7, 24]. The landscape is complex, and failure to resolve the issues around regulatory and bureaucratic deferrals in international studies continues to delay the progress of therapy development. In the rare disease field, this will result in a failure to achieve the targets set by the International Rare Diseases Research Consortium (IRDiRC) [2].

## Methods

The FOR-DMD (Finding the Optimum Steroid Regimen for Duchenne Muscular Dystrophy, ClinicalTrials.gov identifier NCT01603407) study is a multi-centre international study, funded by the NIH, addressing the current equipoise in the use of corticosteroids in Duchenne muscular dystrophy (DMD) [25]. Despite evidence from both randomised trials and cohort studies that corticosteroids are of benefit in DMD [26–28], clinicians continue to be very divided in prescribing corticosteroids in DMD. There are up to 27 different type and dosage regimens currently in routine use, and in some centres, corticosteroids are not prescribed at all [29]. The FOR-DMD study aims to recruit 300 DMD subjects across 40 sites in five different countries (USA, Canada, UK, Germany and Italy) to receive one of the three most commonly prescribed corticosteroid regimens in DMD (daily prednisone, daily deflazacort and intermittent prednisone, 10 days on/10 days off) with the aim to overcome the inconsistencies in corticosteroid prescription. The duration of the study was 3 to 5 years, anticipating that the recruitment target would have been reached in 2 years from study site opening.

The study received funding from the NIH in July 2010 with the understanding that the study would take 6 years: 1 year for study set-up, 2 years for recruitment with each participant being treated and followed up in a blinded manner for 3–5 years depending on when enrolled. Study preparation focussed primarily on contract negotiations between the Sponsor, University of Rochester (recipient of the NIH award) and Newcastle Upon Tyne NHS Hospitals Foundation Trust (NUTH) in the UK (the Sponsor’s Legal Representative in the European Economic Area (EEA) and on drug manufacture. The intent was for recruitment to begin in July 2011; however, the first study sites were not opened for recruitment until January 2013.

Here, we review the process of the FOR-DMD study from funding approval to site activation with the aim to identify the main causes of delay and suggest a checklist of areas of improvements that could predict and potentially overcome these obstacles in future studies.

Although the work on the FOR-DMD study development started much earlier (in 2004), for the purposes of this review, we analysed the set-up process only from the time when funding was approved (July 2010) through to initiation of the 40 sites in the five countries that were originally selected to take part.

**Fig. 1:**
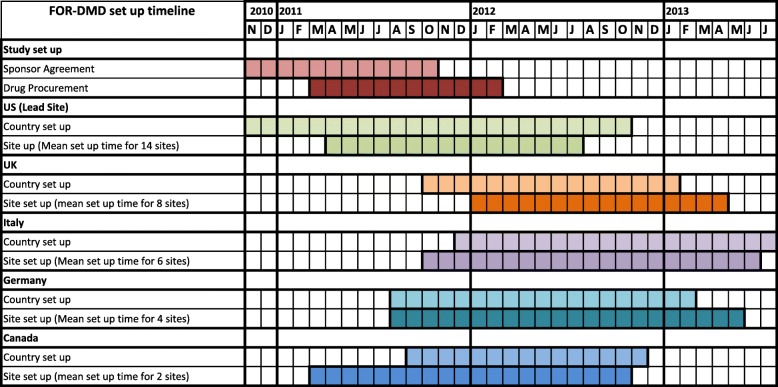
FOR-DMD timeline. Average set-up times for the FOR-DMD study

We analysed the process to set up the collaboration between the Sponsor and the EU Legal Representative, to obtain final study approval in the five countries and in each participating site (Fig. 1). Systematic issues were identified and grouped into (1) study set-up: activities (e.g. drug procurement) that needed to be done before any site in the trial could be opened to recruitment; (2) country set-up: tasks (e.g. competent authority applications) that needed to be done within a given country; and (3) site set-up: activities that needed to be done to finalise individual site approvals.

## Results

### Study set-up challenges

#### Protocol reviews

During the set-up of the FOR-DMD study, the protocol was reviewed 43 times by the NIH and the competent authorities and ethics committees in the five countries involved. In fact, very few modifications were necessary after each single review, but this iterative process delayed the finalisation of the study protocol and the subsequent activities (e.g. submission to regulatory authorities).

#### Budgetary restrictions

The limited resources and budget available for the FOR-DMD study contributed to the delay in study start-up. At the onset, it precluded the possibility to finance CROs to support the regulatory process in the different countries. Additionally, the limited finances allowed no room for site budget negotiation and difficulties with translation of regulatory and study documents (e.g. contracts, questionnaires). Furthermore, there was no contingency for the costs resulting from delays (e.g. additional drug manufacture campaigns to replace expired study drug) or the fluctuation of exchange rates and inflation.

#### Sponsor agreement

FOR-DMD is funded by a US sponsor (NIH) but is also conducted outside the USA. Therefore, according to the EU Clinical Trials Directive, which is the guidance that governs clinical trials in Europe, it is required to have a Legal Representative in the European Economic Area to act as an agent for the sponsor in the event of legal actions. This role was delegated to NUTH in the UK. Clarification of roles and responsibilities between the ‘Sponsor’ and ‘Sponsor’s Legal Representative’ required lengthy repeated discussion between the University of Rochester and NUTH before resolution could be achieved. Involvement of legal representatives for both parties to address risk aversion caused delays in excess of 6 months.

#### Drug procurement

Procurement of the study drug was an additional cumbersome process that created unanticipated delays. Because the UK has much lower infrastructure overheads than the USA, drug supply was coordinated by the EU Legal Representative in the UK. Subsequently, drug procurement fell under EU procurement law, which requires a tendering process: a 30-day advertisement and a 40-day window for the invitation to tender to be returned. Because young study subjects were expected to have difficulty swallowing large, over-encapsulated standard stock tablets, study-specific manufactured tablets were required. This change in the drug preparation, coupled with the need for data on the manufacturing process and on stability testing to feed into the Investigational Medicinal Product (IMP) dossier (including the Investigator Brochure) (required for all competent authority applications), stalled study initiation.

### Country set-up challenges

#### Insurance

Per the EU Clinical Trials Directive, insurance or indemnity to cover the liability of the investigator and sponsor must be secured before commencement of a clinical trial. In the USA and Canada, there are no government mandates for clinical trial liability insurance. Rather, liability falls under the global insurance policy held by each institution. In the EU, insurance and indemnity requirements vary from country to country with no international consistency. For example, Italy requires that each study subject is covered up to €1M, while Germany specifies €500,000. Because insurance requirements were not anticipated, additional time had to be allocated for negotiation around budget. Furthermore, Italy’s financial coverage mandates were modified during the time period between FOR-DMD study planning and trial inception, the compliance with which resulted in even further expense and delay.

#### Translation

An agreement was reached prior study set-up that participating sites were able to accept study documents in English. However, this agreement was set up with the site investigators and did not take into account the requirements of the site’s legal and financial departments. As a result, expenses for translation of study documents into the recruiting countries’ local languages were not fully budgeted in the original grant (i.e. to allow for country- and site-specific modifications), and therefore, final translation of documents was not completed until additional funds were secured from Patient Advocacy Groups (i.e. Italian Telethon). Also, each country had different requirements in terms of document sets that required translation, which was unclear even to the leading site in each country. Until translations were finalised, submission of the study to regulatory authorities in each country was delayed.

#### Lack of regulatory harmonisation

**Fig. 2:**
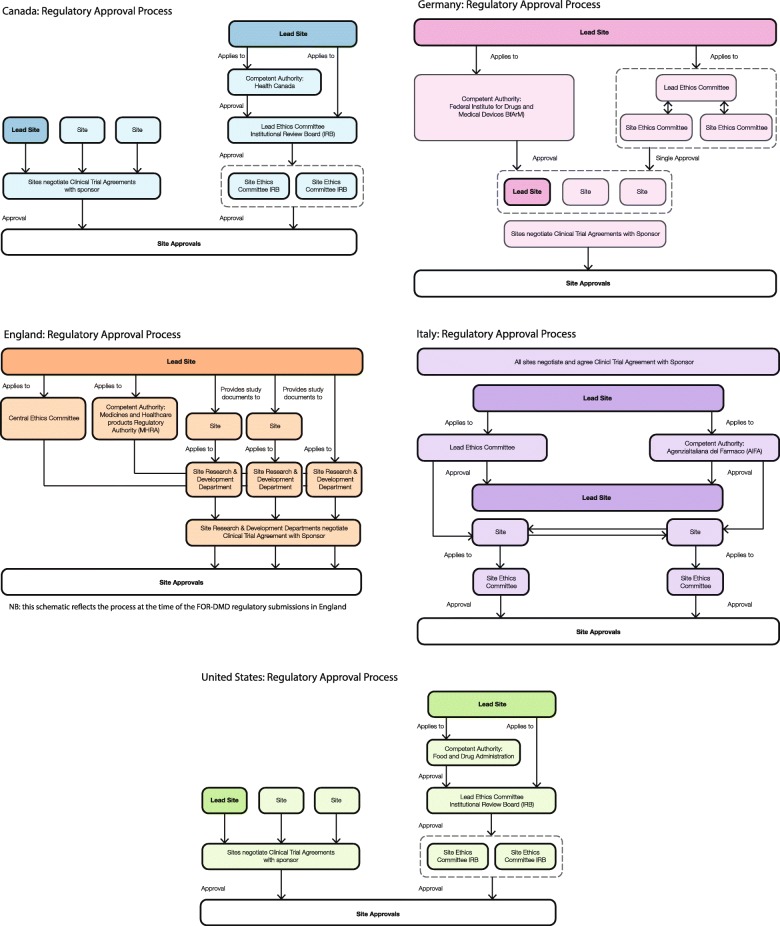
Regulatory approval process. Schematics for approvals in the USA, Canada, Germany, Italy and the UK

In all countries involved in FOR-DMD, there were different regulatory approval processes (including competent authority, ethical and site approvals); this was even the case in countries within the EU. A schematic of clinical trial regulatory approval processes in each participating country is provided in Fig. 2. Each country imposed a distinct and not necessarily overlapping time frame for progress through the regulatory pathway with different conditions at each step. This was compounded by drug manufacturing changes at a ‘study set-up’ level, which meant that essential documents, such as Investigator Brochures, were not available in a timely manner.

### Site set-up challenges

#### Communication

Not using CROs in each country meant that all communications with sites were managed centrally by the study team. Communication in different languages and across multiple time zones was challenging and oftentimes confusing and possibly caused delay in individual site activation.

#### NIH requirements

The US government has specific requirements for studies funded by their institutions, in this case the NIH. These apply not only to entities in the USA but also to non-US-based recipients. All site staff must have proof of Human Subjects Research Training/Good Clinical Practice (GCP). In some EU countries, however, specific GCP certification is not mandatory for clinical staff (i.e. physiotherapists); instead, the clinician only needs to have the relevant training for the tasks that they routinely carry out as part of their job description, regardless of whether that job will be contributing to delivering research. Obtaining GCP compliance from staff at sites, otherwise not subject to this regulation, proved difficult. Moreover, once an individual agreed to obtain the GCP training, they were faced with the challenge of how to accomplish the task. NIH offers a free online course that meets the GCP requirements; however, the course is only offered in English. Although it was a requirement that site Principal Investigators were competent with documents and correspondence in English, language was an issue for other site staff who do not routinely interact in English for clinical and non-clinical duties.

For trials funded by US government organisations (such as the NIH), any site conducting human subjects research must obtain a Federal Wide Assurance (FWA) number, which ensures site compliance with protecting the rights and welfare of human subjects. Additionally, each site must obtain a DUNS (Data Universal Numbering System) number. This number is a unique identifier maintained in a database for entities doing business with (or funded by) the US government. While both of these procedures may be completed online, the systems are often unfamiliar to non-US entities. Registrations of this nature typically must be completed by institution administrators (rather than study staff) who may not be fluent in English. Furthermore, compliance may be an issue in countries where these pieces of information are not typically mandated (particularly obtaining a DUNS number).

Site contracts (subaward agreements) with non-US entities were further slowed by site registration with the System for Award Management (SAM), which combines federal procurement systems with a central contractor registry. SAM registration is required for US entities receiving federal funds (whether directly or as a subaward) but is not currently mandated by the NIH for non-US institutions. However, it is not unusual for the primary award recipient (in this case, the University of Rochester) to require SAM registration for its subaward recipients. SAM registration is completed online, but the submission form is complicated and cumbersome for entities in the EU, which are completely unfamiliar with the format and some of the US-specific terminology. Language barriers, time zone differences and hold times for telephone support (often in excess of 1 h) add to the sites’ burden for completing this task.

#### Site contracts

A UK model Clinical Trial Agreement was used in conjunction with the typical subaward agreement used in the USA, in order to address both EU and US regulations and so that the foreign sites would have a document to review with which (we anticipated) they were already somewhat familiar. However, legal language for clinical trial contracting still caused misunderstanding or conflict with country-specific terminology even within EU sites. This specifically applied to terms like ‘conflict of interest’, ‘intellectual property’ and ‘court of jurisdiction’. Negotiations with Italian sites, for example, continued over several months to determine which court (whether based in the USA or in Italy) would be used in case of subaward agreement dispute (court of jurisdiction). Each time the subaward agreement was reviewed/edited, this added additional delay and cost (e.g. for translation).

## Discussion

The experience of the FOR-DMD study is not unique. Whether in common or rare disease, there have been a number of similar experiences published by investigators working on multi-centre, publically funded clinical trials [5–8, 10, 11]. A lack of regulatory harmonisation inside the EU and between the EU and USA has been reported by many as having the most negative impact in these types of trials, at the centre of this criticism being the EU Clinical Trials Directive [6, 7, 10, 11]. Funder requirements can add a huge additional administrative burden to investigators, particularly when setting up numerous sites globally [5, 6, 8]. There are also differing institutional policies across sites that impact timelines for contracting [5, 8, 10]. Moreover, language barriers exacerbate the ability to overcome these problems quickly [6, 8]. These issues are having a negative impact on multi-centre research.

With this in mind, the current situation appears to be incongruous when considering the ambitious goals set by IRIDIRC for rare diseases [2]. Experienced investigators are struggling to produce results, owing to the fact that the international, multi-centre clinical trials vital to collect enough data for a study to be viable are extremely difficult to set up.

### Initiatives to harmonise approvals

Over the past years, there have been some initiatives to facilitate the process of investigator-led, international study set-up [12–14, 19], but the fruits of these deliberations are not always immediately obvious or operationally in place. In Europe, the highly criticised [18] EU Clinical Trials Directive [30] is due to be replaced by the EU Clinical Trial Regulation, which promises to ease the regulatory burden in the EU countries and provide more harmonisation also for studies that are led and/or funded by the USA [18]. However, these regulations will not be released until 2017, implementation will be gradual, and the document has already been criticised as insufficiently detailed to improve the current situation [31].

To avoid the delays caused through inconsistencies in interpretation of the current EU Clinical Trials Directive, academic networks such as EATRIS and ECRIN have made attempts to provide information about the different regulations and provide international co-ordination [13, 14]. As their application to rare disease to date has been limited, it remains to be seen if their involvement can help to obviate some of the issues encountered in the FOR-DMD trial.

Within the neurology and neuromuscular field in the USA, networks such as NeuroNEXT and CINRG (Cooperative International Neuromuscular Research Group) have tried to harmonise and centrally coordinate the issues of shared contracts and master agreements [20, 32]. However, incentives to use such common formats remain limited and will require concerted action from regulatory and other national authorities.

Although it is widely accepted that harmonisation is needed, and initiatives are being implemented, it will take time for them to be adopted and for improvements to become apparent. This is time that we cannot afford to waste in the development of treatments for rare disease; therefore, interim measures must be made available.

### An interim aid

**Fig. 3:**
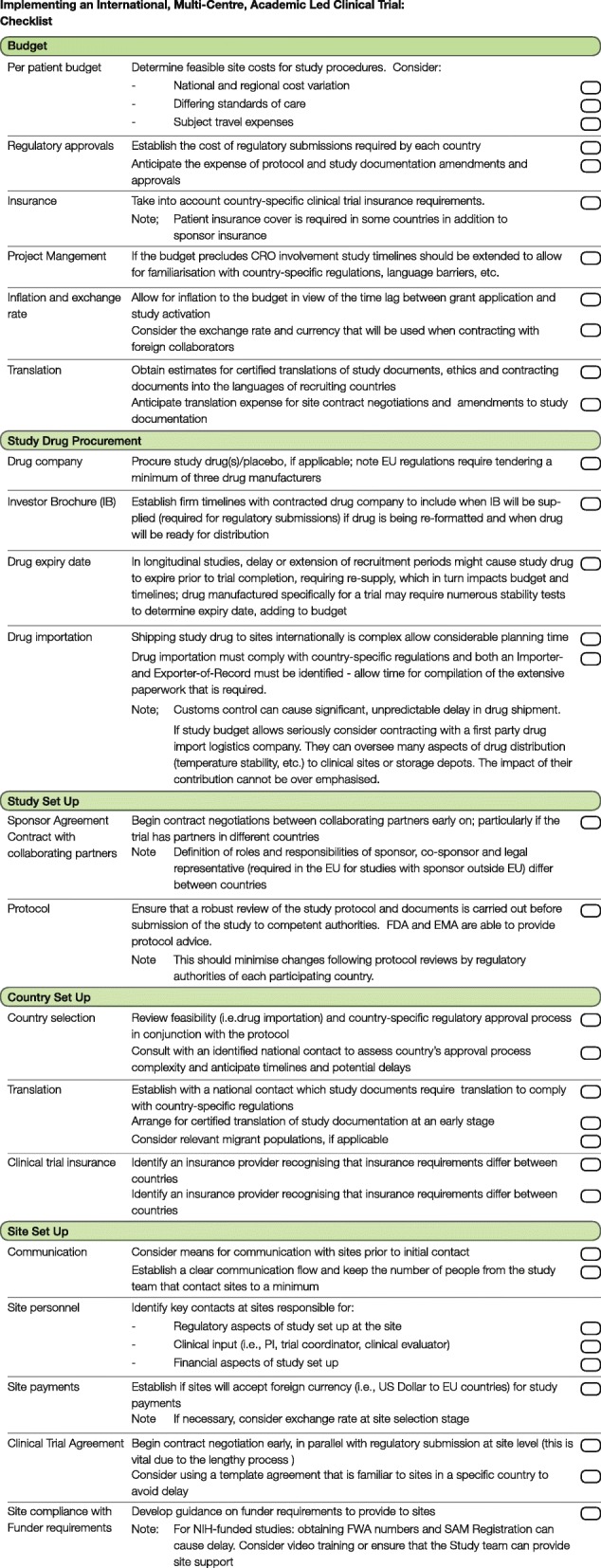
Checklist. A checklist to aid implementing an international, multi-centre, academic-led clinical trial

Using the experiences of setting up the FOR-DMD study, we have developed a checklist of common obstacles and required anticipatory actions (Fig. 3 along with Additional file 1). Shown to be a simple but effective tool within a clinical setting and beyond, the use of a checklist to aid sequencing a complex task has been shown to improve outcomes in clinical practice [33, 34]. This approach therefore not only aims to speed up the set-up of academic-led international clinical trials but also allows investigators to gain a realistic expectation of timelines. The checklist does not aim to change the complex regulatory approval process as it stands, but rather to serve as an interim navigational tool for investigators wanting to develop new treatments using multi-centre clinical trials. We believe that the realistic planning of a clinical trial taking into account the points illustrated in this review will anticipate obstacles and will finally result in an improvement in delivery of clinical trials.

## Conclusion

Our analysis of the set-up of the FOR-DMD study demonstrates that the international regulatory approval system is complex and there is evidence to show that this causes lengthy delays for clinical trial set-up. Given the high need for treatment in rare disease, there is a need for harmonisation. However, more recently, study set-up initiatives have been implemented to facilitate international investigator-led trials, but time is required to operationalise and assess their impact. Perhaps incongruously, there is an extensive push by national and international research organisations to develop new treatments in rare disease, which the current complex system is making difficult to achieve; this is demonstrated by the experience of the FOR-DMD study. It will be up to the national regulatory bodies overseeing research to see if the challenges we have highlighted here can really be addressed. It is not possible for individual groups to change such a complex approval system, but we have used our experience to suggest a checklist and an interim solution, which could represent a useful tool to support academic-led research and will go some way to start bridging the gap between the need to research in rare disease and the regulatory disharmony.

## Additional file

Additional file 1: Checklist supplementary information. Supplementary information to implementing an international, multi-centre, academic-led clinical trial: checklist. (DOCX 115 kb)

## Abbreviations

CINRG: Cooperative International Neuromuscular Research Group
CRO: Contracted research organisations
DMD: Duchenne muscular dystrophy
DUNS: Data Universal Numbering System
EATRIS: European Infrastructure for Translational Medicine
ECRIN: European Clinical Research Infrastructure Network
EEA: European Economic Area
EU: European Union
FOR-DMD: Finding the Optimum Steroid Regimen for Duchenne Muscular Dystrophy
FWA: Federal Wide Assurance
GCP: Good Clinical Practice
IMP: Investigational Medicinal Product
IRDiRC: International Rare Diseases Research Consortium
NeuroNEXT: Network for Excellence in Neuroscience Clinical Trials
NIH: National Institutes of Health
NUTH: Newcastle Upon Tyne NHS Hospitals Foundation Trust
SAM: System for Award Management
TREAT-NMD: Translational Research in Europe - Assessment and Treatment of Neuromuscular Diseases
