# Supplementary material for: A checklist for clinical trials in rare disease: obstacles and anticipatory actions—lessons learned from the FOR-DMD trial
Source: Trials. 2018 May 10;19:291. doi: 10.1186/s13063-018-2645-0 (PMC5968578; doi:10.1186/s13063-018-2645-0)
Supplement: Supplementary file 1 — Checklist supplementary information. Supplementary information to implementing an international, multi-centre, academic-led clinical trial: checklist. (DOCX 115 kb) [file 13063_2018_2645_MOESM1_ESM.docx]

**Additional file 1: Table S1**

**Supplementary information to Implementing an International, Multi-Centre, Academic Led Clinical Trial:**

**Checklist**

| **Pre-Grant Actions** | Developing a thorough budget will require some unpaid pre-grant stage work; however this can result in significant time saving benefits when the grant is awarded. The overall study timelines need to be carefully considered at this stage and should be realistic rather than optimistic. |
| --- | --- |
| **Study Set up** | Uncertainty on timelines for a drug manufacturing company to complete a trial’s investigator brochure can cause major delays with all regulatory applications. Factoring a realistic time for drug procurement and production is an important step to prevent later delays. |
| **Country Set up** | Detailed information regarding the regulatory requirements in different countries should be gathered at early stages and should be taken into account in the selection of the participating countries. The availability of a regulatory contact within a country who speaks the required languages can significantly speed the approval process and therefore the study set up. The identification of this person should therefore be considered as soon as possible. |
| **Site Set Up** | The flow of communication to a site is important and should be decided upon before the site is approached and abided by in order to avoid confusion. Identifying early the relevant contacts at a selected site is also important. This allows early discussion on contract requirements and negotiation. This can be done through the use of a site feasibility assessment, which is an extremely useful tool for avoiding delay. |
